# C-reactive protein gene 1846C>T polymorphism is associated with increased risk and clinical features of lung cancer: a case–control study

**DOI:** 10.1042/BSR20181936

**Published:** 2019-06-20

**Authors:** Chen Chen, Jing-Ni Liu, Jian-Qiang Zhao, Bao Zang

**Affiliations:** 1 Department of Thoracic Surgery, The Affiliated Huaian No.1 People’s Hospital of Nanjing Medical University, Huaian, Jiangsu, China; 2 Department of Burns and Plastic Surgery, The Affiliated Huaian No.1 People’s Hospital of Nanjing Medical University, Huaian, Jiangsu, China

**Keywords:** Chinese population, CRP, lung cancer, risk, single nucleotide polymorphism

## Abstract

Chronic inflammation plays an important role in lung carcinogenesis. Recently, several studies investigated the association of C-reactive protein (CRP) gene 1846C>T polymorphism and lung cancer (LC) risk, but with conflicting findings. In the present study, we conducted this case–control study with 408 LC patients and 472 healthy controls in a Chinese Han population. Genotyping was performed by polymerase chain reaction-restriction fragment length polymorphism (PCR-RFLR) method. Our data found that CRP gene 1846C>T polymorphism increased the risk of LC. Subgroup analyses obtained significant associations among the groups of males, ≥50 years old, smoking, and non-drinkers. Bioinformatics analysis showed that the expression levels of CRP in LC tissues were significantly increased compared with normal tissues. Additionally, the present study found CRP mRNA high expression was associated with worse survival in LC patients. Furthermore, our data indicated that TT genotype of 1846C>T polymorphism was associated with a larger size of tumor and was related with lymphatic metastasis in LC patients. In conclusion, the present study suggests that CRP gene 1846C>T polymorphism is associated with increased risk of LC. CRP gene 1846C>T polymorphism may be a potential marker for the diagnosis of LC.

## Introduction

Lung cancer (LC) is the most common malignancy in the world and is a major cause of cancer-related deaths in developed countries [1]. Up to now, the overall survival rate of this disorder is still remarkedly poor. A host of studies have suggested that tobacco smoking, occupational exposures, and environmental pollution contribute to the risk of LC. However, LC patients may not expose these risk factors [2,3], indicating that some other factors including inflammation may participate in the pathogenesis of LC.

Scientific studies have provided evidence that chronic inflammation plays an important role in the development of cancer carcinogenesis [4]. By the way of cell motility, vascular permeability, and angiogenesis, inflammation promotes the malignant invasion of tissues [5]. Inflammatory cells and cytokines from tumors were reported to associate with tumor growth and metastasis [6]. C-reactive protein (CRP), synthesized predominantly by hepatocytes, is one of the acute-phase proteins. CRP has been reported as a risk factor for many human malignancies [7,8]. Elevated levels of CRP have been shown to increase LC risk [6,9,10]. Recently, some studies explored the association between CRP gene 1846C>T polymorphism and risk of LC [11–13]. However, they yielded contradictory results. To evaluate the role of CRP 1846C>T polymorphism in a Chinese Han population, we designed the present study with 408 LC patients and 472 controls to validate whether CRP 1846C>T polymorphism conferred susceptibility to LC.

## Materials and methods

### Study subjects

The case groups were composed of 408 patients with newly diagnosed LC from the Affiliated Huaian No.1 People’s Hospital of Nanjing Medical University. The inclusion criteria were: (i) they volunteered to participate in the study and signed written consent. (ii) All patients were diagnosed with LC according to the established criteria of clinical, radiologic, and histopathologic reports; (iii) they were not given radiotherapy and/or biological therapy before and during chemotherapy. The exclusion criteria for the LC cases included previous cancer history, lung-related diseases, other types of cancer. We obtained clinical information including age, sex, body mass index (BMI), histological type, and family history of LC by reviewing their medical records.

The 472 healthy controls found at physical examination were recruited from the above-mentioned hospital. The controls had no history of malignancy or any other serious chronic diseases. Informed consent was obtained from each control. The study was approved by the institutional Ethnic Committees of the Affiliated Huaian No.1 People’s Hospital of Nanjing Medical University. The present study was carried out in accordance with the 1964 Declaration of Helsinki.

### Bioinformatics

Oncomine (http://www.oncomine.org), a cancer microarray database and web-based data mining platform, discovers novel targets for therapeutic development, interrogates gene expression profiles and identifies drug and biological interactions. The filtering conditions were as follows: Gene: CRP; Analysis Type: Cancer vs. Normal Analysis; Cancer: Lung Cancer; Sample Type: Clinical Specimen. CRP expression data were from Weiss breast statistics.

The probability of overall survival according to biomarkers was calculated using the Kaplan–Meier plotter (www.kmplot.com) [14]. The median data from Oncomine were divided into two cohorts (high vs. low expression). The CRP gene was uploaded into the database to obtain the Kaplan–Meier survival plot. Log rank *P*-value and hazard ratio (HR) with 95% confidence intervals (CIs) were shown in the figure.

### Determination of genotypes

Genomic DNA was extracted from 2 ml peripheral blood samples using the QIAamp DNA Blood Mini Kit (Qiagen, Hilden, Germany) according to the manufacturer’s recommendation. We evaluated the concentration and quality of extracted DNA using NanoDrop in two OD wavelengths 260 and 280 nm. The DNA was stored at −20°C until use. CRP 1846C>T polymorphism were genotyped using polymerase chain reaction-restriction fragment length polymorphism (PCR-RFLR) method. The primers: 5′-CTTATAGACCTGGGCAGT-3′ (forward) and 5′-GGAGTGAGACATCTTCTTG-3′ (reverse) were synthesized by GenScript Biotechnology (Nanjing, China). The amplification procedure was: denaturing at 95°C for 5 min, followed by 30 PCR cycles (denaturation: 95°C for 30 s, annealing: 56°C for 30 s, extension: 72°C for 45 s) followed by final extension step at 72°C for 10 min. To control the genotyping accuracy, 20 samples for each single nucleotide polymorphism (SNP) was tested in a blind manner. Finally, 2% agarose gel was used to separate PCR products and DNA was visualized by Ethidium Bromide staining.

### Statistical analysis

Demographic and clinical characteristics were calculated using the chi-squared or Student’s *t* test. A goodness-of-fit Chi-squared test was used to test for deviation between observed and expected genotype distributions of CRP 1846C>T polymorphism deviated from the Hardy–Weinberg equilibrium (HWE). The SNP-associated LC risk was expressed as odds ratios (ORs) and 95% CIs. The significant findings were evaluated by calculating false-positive report probability (FPRP). An FPRP threshold of 0.2 and a prior probability of 0.1 were set to detect an OR for a correlation with the tested genotype. FPRP < 0.2 implied a significant relationship. *P*-values less than 0.05 was considered to indicate a significant difference. The Statistical Package for the Social Sciences (SPSS) ver22.0 software package (SPSS Inc., Chicago, IL, U.S.A.) was used for all statistical analyses.

## Results

### Bioinformatics analysis

**Figure 1:**
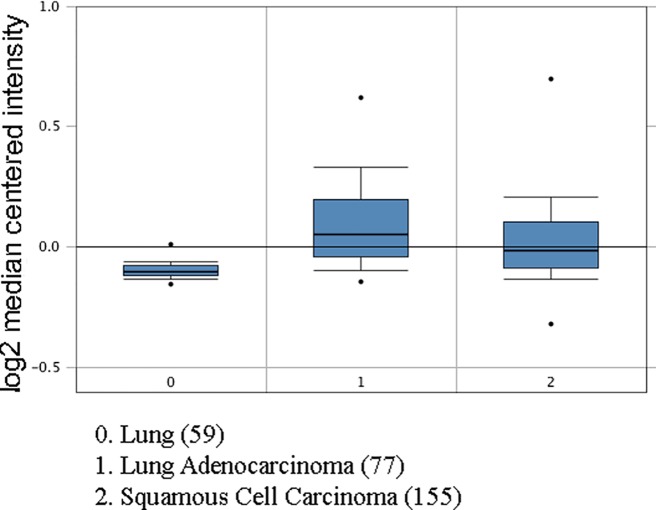
CRP gene expression analysis in LC (Oncomine database) X-axis represents normal (left plot) and cancer tissue (right plot). Y-axis represents the median intensity, 10th and 90th percentile data.

**Figure 2:**
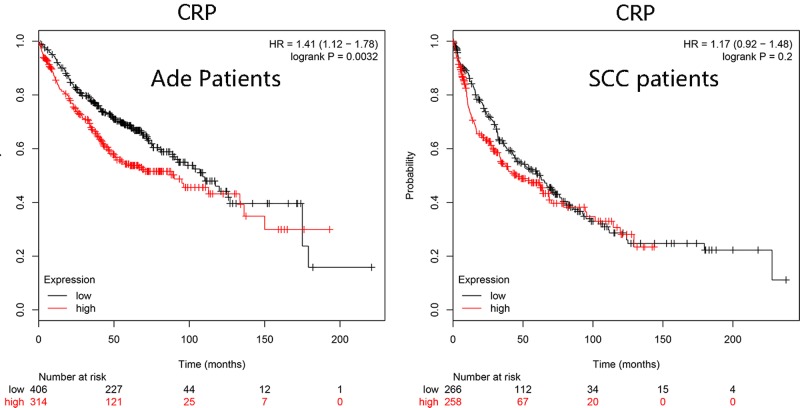
The prognostic value of CRP expression Survival curves were plotted for adenocarcinoma (Ade) patients (*n*=720) and squamous cell carcinoma (SCC) patients (*n*=524).

The elevated expression levels of CRP in both adenocarcinoma and squamous cell carcinoma were observed compared with normal tissues (Figure 1). Then we investigated the association between mRNA expression of CRP and overall survival using a Kaplan–Meier plotter. Survival curves were plotted for adenocarcinoma and squamous cell carcinoma patients. CRP mRNA high expression (Figure 2) was significantly associated with worse survival in adenocarcinoma patients (HR, 1.41 (1.12–1.78), *P*=0.0032).

### Subject characteristics

Baseline characteristics of the study population and their statistical significance are presented in Table 1. LC patients were older (*P*=0.196) and the percent of males was lower in LC patients than control groups (*P*=0.195). The LC patients had significantly higher BMI and percent of individuals with family history of cancer compared with the controls (*P*<0.05). There was a significant difference between the LC cases and the controls in term of smoking and alcohol status. Among the 408 LC cases, 262 (64.22%) were classified as adenocarcinoma, 141 (34.56%) as squamous cell carcinoma, and 5 (1.22%) as other type of LC. No significant deviation from HWE was found for 1846C>T polymorphism in the control groups.

**Table 1:** Patient demographics and risk factors in LC

Variable	Case (*n*=408)	Control (*n*=472)	*P*
Age	62.38 ± 13.17	61.23 ± 12.97	0.196
Sex			0.195
Male	249 (61.03%)	308 (65.25%)	
Female	159 (38.97%)	164 (34.75%)	
BMI	26.68 ± 0.78	25.70 ± 0.89	<0.001
Smoking			0.423
Yes	246 (60.29%)	272 (57.63%)	
No	162 (39.71%)	200 (42.37%)	
Alcohol			0.457
Yes	190 (46.57%)	208 (44.07%)	
No	218 (53.43%)	264 (55.93%)	
Histological type			-
Ade	262 (64.22%)	-	-
SCC	141 (34.56%)	-	-
Other	5 (1.22%)	-	-
Family history of cancer			0.004
Yes	68 (16.67%)	48 (10.17%)	
No	340 (83.33%)	424 (89.83%)	
Tumor size			
≥3 cm	136 (33.3%)		
<3 cm	272 (66.7%)		
Cushing’s syndrome			
Yes	158 (38.7%)		
No	250 (61.3%)		
Lymphatic metastasis			
Yes	66 (16.2%)		
No	342 (83.8%)		

### Association of CRP gene 1846C>T polymorphism with LC risk

Table 2 showed the distribution of genotypes and alleles of the CRP 1846C>T polymorphism in LC cases and controls. The TT genotype was significantly associated with 2.48-fold increased risk of LC in comparison with CC genotype (TT vs. CC, adjusted OR 3.20, 95% CI, 1.87–5.50, *P*<0.001). Furthermore, individuals with (TT+CT) genotypes was also demonstrated to significantly increase the risk for LC (TT vs. CC, adjusted OR 1.64, 95% CI, 1.20–2.66, *P*=0.009). This significant association was also held true in the recessive and allelic models.

**Table 2:** Logistic regression analysis of associations between CRP gene 1846C>T polymorphism and risk of LC

Genotype	Cases* (*n*=408)		Controls* (*n*=472)		OR (95% CI)	*P*	OR (95% CI)^†^	*P*^†^
	*n*	%	*n*	%				
CT vs. CC	192/154	47.2/37.8	216/219	46.0/46.6	1.26 (0.95–1.68)	0.105	**1.41 (1.01, 1.96)**	0.044
TT vs. CC	61/154	15.0/37.8	35/219	7.4/46.6	**2.48 (1.56–3.94)**	<0.001	**3.20 (1.87, 5.50)**	<0.001
TT+CT vs. CC	253/154	62.2/37.8	251/219	53.4/46.6	**1.43 (1.09–1.88)**	0.009	**1.64 (1.20, 2.26)**	0.002
TT vs. CT+CC	61/346	15.0/85.0	35/435	7.4/92.6	**2.19 (1.41–3.40)**	<0.001	**2.68 (1.61, 4.45)**	<0.001
T vs. C	314/500	38.6/61.4	286/654	30.4/69.6	**1.44 (1.18–1.75)**	<0.001		

^*^The genotyping was successful in 407 cases and 470 controls.

^†^Adjusted for sex, age, BMI.

Bold values are statistically significant (*P*<0.05).

Stratified analyses were performed according to sex, age, smoking, alcohol, and family history of cancer (Table 3). Subgroup analysis of sex indicated that this SNP correlated with increased risk of LC among males in the codominant, recessive, and dominant models and this association held true for ≥50 years old, smokers, non-drinkers, and individuals with BMI ≥ 25.

**Table 3:** Stratified analyses between CRP 1846C>T polymorphism and the risk of LC

Variables	*rs1205* (case/control)				OR (95% CI); *P*			
	CC	CT	TT	CT+TT	CT versus CC	TT versus CC	TT versus CT+CC	CT+TT versus CC
Sex								
Male	91/144	117/139	40/23	157/162	1.33 (0.93–1.91); 0.118	**2.75 (1.55–4.90); <0.001**	**2.37 (1.37–4.07); 0.002**	**1.53 (1.09–2.16); 0.014**
Female	63/75	75/77	21/12	96/89	1.16 (0.73–1.84); 0.530	2.08 (0.95–4.57); 0.067	1.93 (0.91–4.06); 0.085	1.28 (0.83–2.00); 0.268
Age (years)								
<50	17/31	39/43	8/6	47/49	1.27 (0.75–2.16); 0.376	1.65 (0.69–3.96); 0.262	1.45 (0.64–3.32); 0.377	1.33 (0.80–2.21); 0.272
≥50	137/188	153/173	53/29	206/202	1.26 (0.90–1.77); 0.172	**2.89 (1.66–5.02); <0.001**	**2.56 (1.51–4.32); <0.001**	**1.48 (1.08–2.04); 0.016**
Smoking								
Yes	83/125	124/126	39/20	163/146	**1.48 (1.02–2.15); 0.038**	**2.94 (1.60–5.38); <0.001**	**2.36 (1.34–4.18); 0.003**	**1.68 (1.18–2.40); 0.004**
No	71/94	68/90	22/15	90/105	1.00 (0.64–1.55); 0.999	1.94 (0.94–4.01); 0.072	1.94 (0.97–3.88); 0.060	1.14 (0.75–1.72); 0.553
Alcohol								
Yes	71/89	90/96	28/22	118/118	1.17 (0.77–1.80); 0.456	1.60 (0.84–3.02); 0.152	1.46 (0.81–2.66); 0.212	1.25 (0.84–1.88); 0.272
No	83/130	102/120	33/13	135/133	1.33 (0.91–1.95); 0.141	**3.98 (1.98–8.00); <0.001**	**3.43 (1.76–6.70); <0.001**	**1.59 (1.10–2.29); 0.013**
Family history of cancer								
Yes	26/29	34/15	8/4	42/19	**2.53 (1.13–5.66); 0.024**	2.23 (0.60–8.28); 0.231	1.47 (0.42–5.18); 0.552	**2.47 (1.16–5.26); 0.020**
No	128/190	158/201	53/31	211/232	1.17 (0.86–1.59); 0.323	**2.54 (1.54–4.17); <0.001**	**2.34 (1.46–3.74); <0.001**	**1.35 (1.01–1.81); 0.044**
BMI								
<25	1/45	3/49	1/6	4/55	2.76 (0.28, 27.45); 0.388	7.50 (0.41, 136.27); 0.173	3.92 (0.38,40.72); 0.253	3.27 (0.35, 30.31); 0.297
≥25	153/174	189/167	60/29	249/196	1.29 (0.95, 1.74); 0.100	**2.35 (1.44, 3.85); 0.001**	**2.06 (1.29, 3.29); 0.002**	**1.45 (1.09, 1.93); 0.012**

Bold values are statistically significant (*P*<0.05).

### Relationship between CRP 1846C>T polymorphism and clinical features of LC

We also investigated the effect of CRP 1846C>T polymorphism on the different clinical features of LC patients (Table 4). Our results indicated that TT genotype of 1846C>T polymorphism was associated with a larger size of tumor in LC patients (TT vs. CC, OR 1.93, 95% CI, 1.05–3.55, *P*=0.033). In addition, we found that TT genotype was related with lymphatic metastasis (TT vs. CC, OR 1.88, 95% CI, 1.03–3.45, *P*=0.040). However, there were no significant associations between this SNP and other clinical parameters of LC patients (Cushing’s syndrome and adenocarcinoma).

**Table 4:** The associations between CRP 1846C>T polymorphism and clinical characteristics of LC patients

Characteristics	Genotype distributions			
	CC	CT	TT	CT+TT
Tumor size (cm)				
<3/≥3	47/107	66/126	28/33	94/159
OR (95% CI); *P*-value	1.00 (reference)	1.19 (0.76, 1.88); 0.447	**1.93 (1.05, 3.55); 0.033**	1.35 (0.88, 2.06); 0.172
Cushing’s syndrome				
Yes/No	27/127	27/165	12/49	39/204
OR (95% CI); *P*-value	1.00 (reference)	0.77 (0.43, 1.38); 0.377	1.15 (0.54, 2.45); 0.714	0.90 (0.53, 1.54); 0.699
Lymphatic metastasis				
Yes/No	50/104	72/120	29/32	101/152
OR (95%CI); *P*-value	1.00 (reference)	1.25 (0.80, 1.95); 0.330	**1.88 (1.03, 3.45); 0.040**	1.38 (0.91, 2.11); 0.132
Adenocarcinoma				
Yes/No	103/51	118/74	40/21	158/95
OR (95%CI); *P*-value	1.00 (reference)	0.79 (0.51, 1.23); 0.297	0.94 (0.50, 1.76); 0.854	0.82 (0.54, 1.26); 0.366

Bold values are statistically significant (*P*<0.05).

The FPRP values for 1846C>T polymorphism at different *P* levels are summarized in Table 5. At the level of 0.1, some FPRPs were all <0.20, indicating the significant associations between TNF-α rs361525 polymorphism and GC risk were noteworthy under the homozygous, recessive, and allelic models (Table 5).

**Table 5:** FPRP values for associations between CRP 1846C>T polymorphism and risk of LC

Variables	OR (95% CI)	*P*-value	Power	Prior probability				
				0.25	0.1	0.01	0.001	0.0001
TT vs. CC	**2.48 (1.56–3.94)**	<0.001	0.997	0.004	0.013	0.122	0.584	0.934
TT+CT vs. CC	**1.43 (1.09–1.88)**	0.009	0.926	0.053	0.144	0.650	0.949	0.995
TT vs. CT+CC	**2.19 (1.41–3.40)**	<0.001	0.991	0.005	0.015	0.146	0.633	0.945
T vs. C	**1.44 (1.18–1.75)**	<0.001	0.995	0.005	0.014	0.133	0.607	0.939

## Discussion

In the present study, we found that CRP 1846C>T polymorphism was associated with increased risk of LC. Subgroup analyses yielded significant associations among the groups of males, ≥50 years old, smoking, and non-drinkers.

The association between CRP levels and cancers has been comprehensively explored. A host of studies provided compelling evidence that high CRP levels were associated with a poor prognosis in cancer patients, including LC [15–18]. Chaturvedi et al. [11] found elevated CRP levels were associated with increased LC risk. A host of studies investigated the association between CRP gene polymorphism and cancer risk. Kito et al. [19] found CRP 1846C>T polymorphism was related to lymph node metastasis and severe lymphatic invasion in endometrial cancer. Other studies demonstrated 1846C>T polymorphism as a novel predictor of lymph node metastasis in invasive breast cancer [20], submucosal thoracic esophageal squamous cell carcinoma (ESCC) [21], indicating the close association between this SNP and cancer risk. Motoyama et al. [22] first explored the association between 1846C>T polymorphism and thoracic esophageal cancer; however, they did not obtain any significant findings regarding this SNP. Siemes et al. from Netherlands also failed to identify this association in colorectal cancer [23]. However, several studies obtained a significant association between CRP 1846C>T polymorphism and colorectal cancer risk [23–27]. It is worth noting that a meta-analysis did not identify any association between CRP 1846C>T polymorphism and the risk of colorectal cancer [28]. A study from Japan showed the frequency of the CRP 1846C>T polymorphism among the LC patients did not differ from the frequency among the hospital controls [12]; however, they uncovered the CRP 1846T/T genotype was associated with a poor prognosis in LC patients. Another Caucasian study [11] indicated that 1846C>T polymorphism was not associated with LC risk. Nevertheless, they showed this SNP was associated with CRP levels, and increased CRP levels were associated with increased LC risk. Similar results were replicated in a Chinese study [13]. Different sample sizes, genetic heterogeneity, clinical heterogeneity, and diverse lifestyles and environments may explain these conflicting results.

In the present study, we found CRP 1846C>T polymorphism was associated with increased risk of LC, which is in consistence with the finding of a meta-analysis showing an increased risk for overall cancer [29]. Furthermore, this is the first study uncovering an association between CRP 1846C>T polymorphism and LC risk. Heikkila et al. [30] also provided some evidence for an association of a small number of CRP-associated SNPs with the overall cancer and LC risk. A previous study showed that an SNP of CRP gene was associated with CRP levels at the candidate gene level [31]. Furthermore, bioinformatics analysis of this study showed that the increased expression levels of CRP in LC tissues compared with normal tissues. Additionally, we found CRP mRNA high expression was associated with worse survival in LC patients (Figure 2). Thus, we assumed that 1846C>T polymorphism may participate in the regulation of CRP levels, therefore contributing to increased risk for LC patients. In the subgroup analysis, the present study obtained positive findings among the groups of males, ≥50 years old, smoking, and non-drinkers. We think these findings could be explained by the opinion that susceptible individuals are more likely to expose to these risk factors. Clinically, males, older individuals, and smokers are regarded as high-risk LC groups. Another explanation is that these factors interact with CRP 1846C>T polymorphism, which may be associated with increased risk of LC patients. However, given the limited sample sizes of stratification analysis, we should interpret these results with caution.

Some potential limitations of the present study should be noted. One, because it is a retrospective study, selection bias could not be completely avoided. Two, the sample size of stratification analysis in the present study was not large enough. Three, LC is a heterogeneous disease; thus, we should investigate the interaction between SNPs and environmental factors. Four, only one SNP in CRP gene was investigated; additional CRP gene variants should be studied. Five, bioinformatics analyses were just made from database and not really validated with any sort of experiments. At last, we could not explain why CRP substratification for adenocarcinoma was more significant than squamous cell carcinoma. To sum up, CRP 1846C>T polymorphism is associated with increased risk of LC patients in this Chinese Han population. CRP 1846C>T polymorphism will be a potential marker for the diagnosis of LC. More studies with larger sample sizes in other races are warranted to validate this association.

## Abbreviations

BMI: body mass index
CI: confidence interval
CRP: C-reactive protein
FPRP: false-positive report probability
HR: hazard ratio
HWE: Hardy–Weinberg equilibrium
LC: lung cancer
OR: odds ratio
SNP: single nucleotide polymorphism
